# A genomic mutation spectrum of collecting duct carcinoma in the Chinese population

**DOI:** 10.1186/s12920-021-01143-2

**Published:** 2022-01-03

**Authors:** Huaru Zhang, Xiaojun Lu, Gang Huang, Meimian Hua, Wenhui Zhang, Tao Wang, Liqun Huang, Ziwei Wang, Qing Chen, Jing Li, Qing Yang, Guosheng Yang

**Affiliations:** 1 The Second School of Clinical Medicine, Southern Medical University, Guangzhou, 510515 China; 2 Department of Urology, Guangdong Second Provincial General Hospital, Guangzhou, 510317 China; 3 Department of Urology, Shanghai East Hospital, Tongji University School of Medicine, Shanghai, 200120 China; 4 Department of Urology, the First Affiliated Hospital, Naval Military Medical University, Shanghai, 200433 China; 5 Department of Bioinformatics, Center for Translational Medicine, Second Military Medical University, Shanghai, 200433 China; 6 Department of Urology, the First Affiliated Hospital of Zhengzhou University, Zhengzhou, 450052 Henan China; 7 Department of Urology, Shanghai Ninth People’s Hospital, Shanghai Jiaotong University School of Medicine, Shanghai, 200011 China

**Keywords:** Collecting duct carcinoma, Somatic mutations, Copy number variants, *CDKN2A*

## Abstract

**Background:**

Renal collecting duct carcinoma (CDC) is a rare and lethal subtype of renal cell carcinoma (RCC). The genomic profile of the Chinese population with CDC remains unclear. In addition, clinical treatments are contradictory. In this study, we aimed to identify the genomic mutation spectrum of CDC in the Chinese population.

**Methods:**

Whole-exome sequencing was performed using the Illumina Novaseq™ 6000 platform. MuTect2 detects single-nucleotide variants (SNVs) and small scale insertions/deletions (INDELs). The identified mutations were annotated with ANNOVAR and validated by Sanger sequencing. Control-FREEC was used to detect copy number variation (CNV), and GISTIC was applied to detect frequently mutated altered regions. These data were compared with associated The Cancer Genome Atlas cohorts.

**Results:**

Ten normal-matched CDC patients were included. The mean tumour mutation burden was 1.37 Mut/Mb. Six new recurrent somatic mutated genes were identified, including *RBM14*, *MTUS1*, *GAK*, *DST*, *RNF213* and *XIRP2* (20% and 2 of 10, respectively), and validated by Sanger sequencing. In terms of common mutated genes, *SETD2* was altered in both CDC and other RCC subtypes but not in bladder urothelial carcinoma (BLCA); *CDKN2A* was a driver gene in both CDC (SNV: 10%, 1 of 10) and BLCA but not in other RCC subtypes. Next, 29 amplifications and 6 deletions of recurrent focal somatic CNVs were identified by GISTIC2.0, which displayed differences from kidney renal clear cell carcinoma (KIRC), kidney renal papillary cell carcinoma (KIRP) and BLCA cohorts. Of note, *CDKN2A* (CNV alteration: 30%, 3 of 10) and *CDKN2A-AS1* were the only overlapping genes of these four cohorts. Importantly, the *CDKN2A* mutation in our cohort differed from previous studies in urinary carcinomas. Moreover, *CDKN2A-*altered cases had significantly worse overall survival than wild-type cases in both KIRC and KIRP cohorts. In addition, the most frequently altered genomic pathway of our CDC cohort was the *CDKN2A*-mediated p53/RB1 pathway.

**Conclusions:**

Our study offers the first genomic spectrum of the Chinese population with CDC, which differs from that of the Western population. The altered *CDKN2A*-mediated p53/RB1 pathway might provide new insight into potential therapeutic targets for CDC patients.

**Supplementary Information:**

The online version contains supplementary material available at 10.1186/s12920-021-01143-2.

## Background

Collecting duct carcinoma (CDC) is a rare and lethal subtype of renal cell carcinoma [1] that is still mainly diagnosed based on pathological examination [2, 3]. Approximately half of CDC patients are initially diagnosed at an advanced stage with metastatic symptoms of the lymph node, bone, lung, or liver, and most die within 1–3 years [4, 5]. Moreover, the clinical treatments are contradictory. The kidney cancer part of the National Comprehensive Cancer Network (NCCN) guidelines recommend platinum-based chemotherapies due to some shared biological features with urothelial carcinoma [6]. However, 23 metastatic CDC patients treated with gemcitabine plus cisplatin or carboplatin showed only an objective response rate (ORR) of 26% and an overall survival (OS) of 10.5 months [7]. In addition, a systemic therapy for CDC, renal cell carcinoma (RCC), has been proposed according to a transcriptome sequencing study [8], yet the outcomes are unsatisfactory. Combined chemotherapies showed an ORR of 30.8% and an OS of 12.5 months [9], which was similar to single chemotherapies [10]. Targeted therapies for metastatic CDC have little clinical benefit [11], and no response to immunotherapy has been observed [4, 12].

Therefore, additional comparative studies are urgently needed to better distinguish the dominant molecular signature between CDC and other RCC subtypes or urothelial carcinomas, providing new insight into potential prognostic and therapeutic targets.

There are a few genomic studies to uncover genetic alterations in CDC patients. Seventeen advanced or metastatic American patients with CDC identified 36 genomic alterations by targeted next-generation sequencing of established rearrangement- and cancer-related genes, e.g., *NF2* (29%), *SETD2* (24%), *SMARCB1* (18%), *CDKN2A* (12%), *PIK3CA* (6%), *PIK3R2* (6%), *FBXW7* (6%), *BAP1* (6%), *DNMT3A* (6%), *VHL* (6%) and *HRAS* (6%). In the study, alterations in *FH* and *SMARCB1* occurred in a mutually exclusive manner to *NF2* alterations [13]. Moreover, a multicentre copy number variant calling study of 29 German CDC patients revealed recurrent DNA losses at 1p, 8p, 9p and 16p and gains at 13q [14]. Whole-exome sequencing (WES) of four normal-matched American CDC patients identified only one recurrent somatic single-nucleotide variant [15]. In addition, an integrative transcriptomic study of 17 CDC patients identified that CDC may originate from the distal convoluted tubules of the nephron. Moreover, CDC is considered not only a metabolic disease but also an oxidoreductase activity and pyruvate metabolism [16]. These studies have mainly focused on Western populations, whereas the genomic profile of CDC patients in the Chinese population remains unclear.

Therefore, in this study, we performed deep whole-exome sequencing of 10 paired CDC patient tumour tissues that matched normal kidney tissues to improve our understanding of the genomic profile of Chinese patients with CDC and compared the results with other RCC subtypes and bladder urothelial carcinoma. We found that CDC is not only characterised as a unique type of solid tumour but also shares some specific molecules with other RCC subtypes and urinary tract carcinoma. The *CDKN2A* alteration-mediated *p53*/*RB1* pathway might provide new insight into potential prognostic and therapeutic targets for these patients.

## Materials and methods

### Study design and samples information

Patients were collected retrospectively from the First Affiliated Hospital (Changhai Hospital), Naval Military Medical University. Paired tumour and normal formaldehyde-fixed paraffin-embedded (FFPE) samples were obtained prior to any treatment. Pathological diagnoses were reconfirmed by two experienced uropathologists. Patients with urothelial carcinoma involving the upper tract, papillary RCC, clear cell carcinoma RCC, chromophobe RCC, unclassified RCCs and other malignant tumours were excluded.

Ethics approval was obtained from the institutional review board of Changhai Hospital (CHEC2021-064). The study was conducted in accordance with the Helsinki Declaration of 1975, as revised in 1983, and the Good Clinical Practice guidelines. All research participants or their legal representatives signed informed consent forms for participation in the research.

### DNA extraction and quantification

Genomic DNA was extracted using QIAamp DNA FFPE Tissue Kit (QIAGEN). DNA quality and yield were measured and assessed using a Qubit fluorometer and Qubit dsDNA HS Assay Kit (Thermo Fisher) following the manufacturer’s protocol.

### WES library generation and sequencing

Before library generation, genomic DNA was fragmented by sonication to a median size of 350 bp. Then, the KAPA hyperprep kit (Roche) was used for library preparation, and xGen® Hybridization and Wash Kit (IDT) was used for exome capture before sequencing. Next, genomic DNA fragments were end-repaired, ligated with Illumina sequencing adapters, and amplified. Finally, DNA libraries were subjected to WES using the Illumina Novaseq™ 6000 platform (2 × 150-bp paired-end reads).

### Sequencing data analysis

Paired-end reads were quality checked by FastQC (v0.11.9) and processed to high quality using Trimmomatic [17] (v0.36, parameters: SLIDINGWINDOW: 4: 15, LEADING: 3, TRAILING: 3, ILLUMINACLIP: adaptor.fa: 2: 30: 10: 8: ture, MINLEN: 36) to remove adapters and perform trimming. The reads were aligned to Human Genome Reference Consortium build 38 (GRCh38) using Burrows-Wheeler Aligner (BWA-MEM) v0.7.8.

### Somatic mutations calling

Somatic mutations, including single-nucleotide variants (SNVs) and small-scale insertions/deletions (INDELs), were detected using the Mutect2 pipeline in Genome Analysis Toolkit (GATK, v4.1.9.0). ANNOVAR [18] was applied to annotate filtered variant call format files using multiple annotation databases. Briefly, mutations in segmental duplications (genomicSuperDups) or repetitive elements (RepeatMasker) were removed. Non-synonymous exonic mutations with minor allele frequency > 5% in the 1000 Genome Project, Exome Aggregation Consortium database with allele frequencies in East Asia (EAS), dbSNP 138, or exome sequencing project (ESP) were removed; all COSMIC variants were retained. Mutations with a variant allele frequency (VAF) greater than 0.03 after filtering were reviewed manually using integrated genomics viewer (IGV). Finally, mutations within the blacklist [19] were also filtered and removed. Next and importantly, recurrent mutated genes were experimentally validated by Sanger sequencing.

Further sequencing analyses, including the significantly mutated genes (SMGs), mutation signature pattern and tumour mutation burden, were also performed. Briefly, the prepared mutation annotation format file was analysed to determine SMGs using MutSigCV (v2.0), with a cut-off value of *p* < 0.05. The deconstructSigs R package [20] was adopted to calculate the ratios of 30 types of defined COSMIC mutation signatures [21] in each sample. Tumour mutation burden was defined as in our previous study [22], with 34.2 Mb of exonic region.

### Somatic copy number alterations calling

Control-FREEC [23] (v.11.5) was used to detect genomic segments with somatic copy number variants (CNVs) under default parameters. The GISTIC2.0 [24] algorithm was applied to detect recurrently amplified and deleted genomic regions with the following modified parameters: -ta 0.1, -td 0.1, -js 100; -broad 1; -brlen 0.7; -conf 0.95; -genegistic 1; and -savegene 1.

### Comparison of mutation landscapes and pathways across CDC and associated TCGA cohorts

The integrated SNV and CNV sequencing data of associated The Cancer Genome Atlas (TCGA) cohorts, including kidney renal clear cell carcinoma (KIRC), kidney renal papillary cell carcinoma (KIRP), and bladder urothelial carcinoma (BLCA), were downloaded from cBioPortal (https://www.cbioportal.org). Next, RCircos [25] and Venn diagrams were applied to visualise and compare the different distributions of the above CNV-based genes in our CDC, KIRC, KIRP and BLCA cohorts.

In addition, the mutation frequency of all key genes in 10 common and 3 specific cancer-related pathways related to SNV and CNV were used to compare the mutation landscape and pathway enrichment across these four study cohorts and to determine the putative critical pathway in our CDC cohort. The components of all the altered genes in each pathway were also calculated.

### *CDKN2A* mutation spectrum in CDC and associated TCGA cohorts

Available SNV, CNV and overall survival data of *CDKN2A* for KIRC, KIRP, BLCA and breast cancer (BRCA) patients were downloaded from cBioPortal. The percentages of patients with SNVs, CNV alterations and wild-type *CKDN2A* were summarised, and the mutation spectrum of *CDKN2A* in these cohorts was analysed.

## Results

### Patient clinical information

**Fig. 1:**
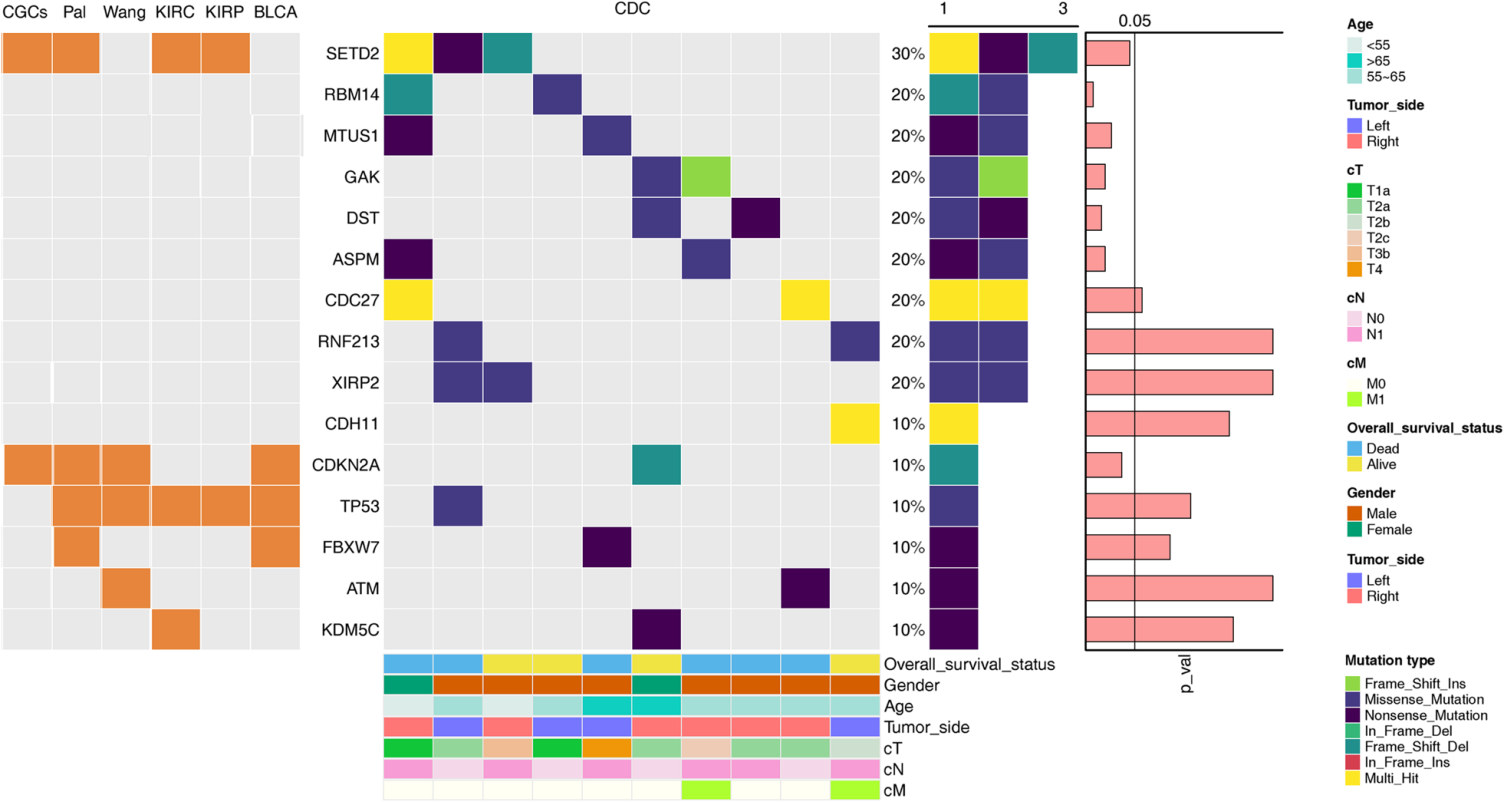
The landscape of somatic mutations. Left panel: The presence of 15 somatically altered genes in Cancer Gene Census and two previous collecting duct carcinoma (CDC) studies (Pal et al. and Wang et al.) as the heatmap on the left. Right panel: Each column represents an individual tumour patient (n = 10). Mutation profiles of the top 10 genes detected by maftools and five frequently mutated CDC-related genes using genome analysis toolkit (GATK) are displayed. Mutation frequencies and numbers are shown in the middle, and seven related clinicopathological characteristics for all 10 patients are shown at the bottom, including overall survival status, sex, age, tumour location, clinical T stage, clinical N stage, and clinical M stage. The *p* values of the mutated genes detected by MutSigCV2.0 are plotted on the right

Ten CDC patients with matched tumour and normal renal tissues were included, and their clinical information is summarised in Fig. 1 and Additional file 1: Table S1. Of these patients, eight were male and two female. The median age was 57.3 years old (range: 33–67). The ratio of left to right tumour side was 1: 1.5. Two patients were diagnosed with a high clinical T stage (T3 and T4), though most of the others were at T2. More than half of the patients had local lymph node infiltration, and two patients had distant metastases.

### The landscape of somatic mutations

The average sequencing depth was 534.78× (range: 302.30–794.62×) in tumours and 128× (range: 89.27–195.13×) in normal samples (Additional file 2: Table S2). We identified 471 filtered SNVs, including 359 nonsynonymous mutations, 31 stop-gain mutations, 2 stop-loss mutations, 50 frameshift INDELs and 29 nonframeshift INDELs (Additional file 3: Table S3). The median mutation burden of each sample was 1.37 Mut/Mb (range: 0.41–2.54).

As shown in Fig. 1, *SETD2* was the most frequently mutated gene (30%). Some common mutated genes previously reported in CDC were also identified, including *CDKN2A*, *TP53*, *FBXW7*, *ATM* and *KDM5C* (10%). Moreover, eight novel recurrent somatic mutated genes were detected, including *RBM14*, *MTUS1*, *GAK*, *DST*, *ASPM*, *CDC27*, *RNF213* and *XIRP2* (20%). To validate these findings, Sanger sequencing was performed. Seven of nine recurrently mutated genes in our study were successfully validated; *ASPM* and *CDC27* were not (Additional files 4 and 5: Figures S1 and S2). *RBM14*, *GAK*, *DST* and *XIRP2* were selected to validate different variants of the same gene. However, some variant validations failed; variant allele frequencies (VAFs) were 11.0% (*RBM14*), 11.2% (*DST*) and 11.2% (*XIRP2*) (Additional file 4: Figure S1).

Next, compared with previous studies with CDC and associated TCGA cohorts, including KIRC, KIRP and BLCA, *TP53* mutation was detected in all six studies. *SETD2* was altered in Pal et al.’s study and our study and in KIRC and KIRP but not in BLCA. *CDKN2A* was a driver gene in both CDC and BLCA but not in KIRC and KIRP.

Further analysis identified *SETD2*, *RBM14*, *MTUS1*, *GAK*, *DST*, *ASPM* and *CDKN2A* as SMGs. However, *SETD2* is the only SMG in Cancer Gene Census. The mutation signature patterns of each patient were examined, and those of 1, 3, 5, 8 and 9 were the most common (Additional file 6: Figure S3).

### The landscape of copy number variants

**Fig. 2:**
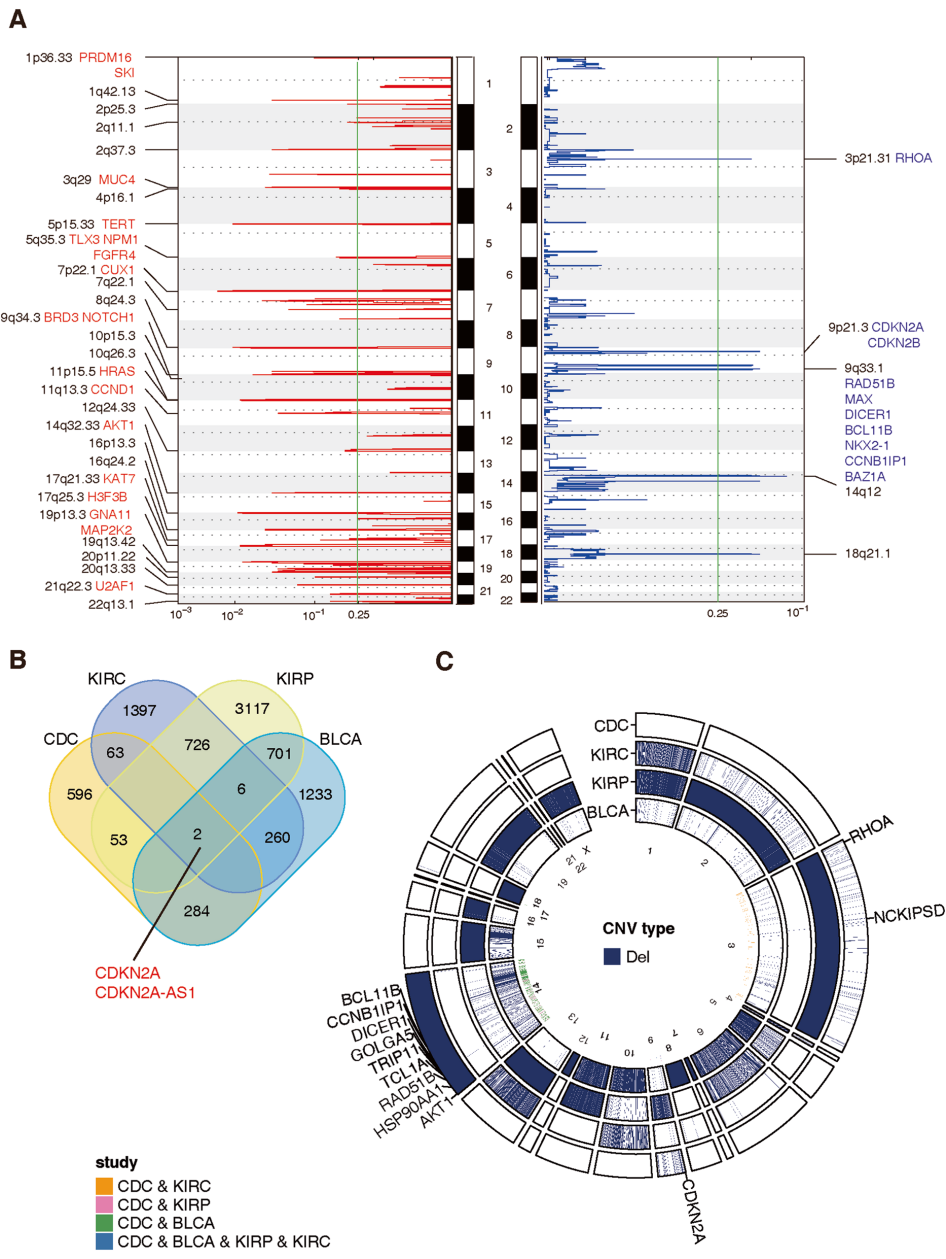
The landscape of copy number variants (CNVs). **A** Left: Significant (q < 0.25) recurrent focal amplified CNVs detected by Control-FREEC along all autosomes using GISTIC 2.0 are shown. Recurrent putative cancer driver genes of each SCNA are also highlighted. Right: Significant (q < 0.25) recurrent focal deleted CNVs detected by Control-FREEC along all autosomes using GISTIC 2.0 are shown. Recurrent putative cancer driver genes of each SCNA are also highlighted. **B** Venn diagram of the four included cohorts using the CNV-based deleted genes. **C** Rcircos results of the four included cohorts using the CNV-based deleted genes. KIRC, clear cell renal cell carcinoma; KIRP, papillary renal cell carcinoma; BLCA, bladder carcinoma; CDC, collecting duct carcinoma

We analysed somatic CNVs using Control-FREEC mutation-calling software. GISTIC2.0 analysis identified 29 amplified and 6 deleted recurrent focal CNVs (Fig. 2A). In terms of driver genes in Cancer Gene Census, focal amplified regions implicated the oncogenes *PRDM16* and *SKI* at 1p36, *MUC4* at 3q29, *TERT* at 5P15, *TLX3*, *NPM1*, *FGFR4* and *FLT4* at 1q35, *CUX1* at 7p22, *BRD3* and *NOTCH1* at 9q34, *HRAS* at 11p15, *CCND1* at 11q13, *AKT1* at 14q32, *KAT7* at 17q21, *H3F3B* at 17q25, *GNA11*, *TCF3*, *MAP2K2*, *FSTL3* and *SH3GL1* at 19p13, and *U2AF1* at 21q22. However, only *HRAS* has been previously reported in CDC [13]; it belongs to the Ras oncogene family and induces GTPase activity [26]. Focally deleted regions identified the tumour-suppressor genes *RHOA* at 3p21, *CDKN2A* and *CDKN2B* at 9p21, *RAD51B*, *MAX*, *DICER1*, *BCL11B*, *NKX2-1*, *CCNB1IP1* and *BAZ1A* at 9q33.

At the CNV-based gene level, only *CDKN2A* and *CDKN2A-AS1* were found using the deleted genes (Fig. 2B; Additional file 7: Table S4). The overlapping deleted genes were enriched on chromosomes 3 and 14 (Fig. 2C). However, no common genes were found among amplified genes (Additional file 8: Fig. S4A; Additional file 9: Table S5), and overlapping amplified genes were enriched on chromosomes 5 and 17 (Additional file 8: Fig. S4B).

### *CDKN2A* alteration is important in CDC and associated TCGA cohorts

Since *CDKN2A* is a key mutated gene according to the somatic mutation and copy number alteration landscapes, we further explored *CDKN2A* alteration in CDC and associated TCGA cohorts, including BLCA, KIRC, KIRP and BRCA.

**Fig. 3:**
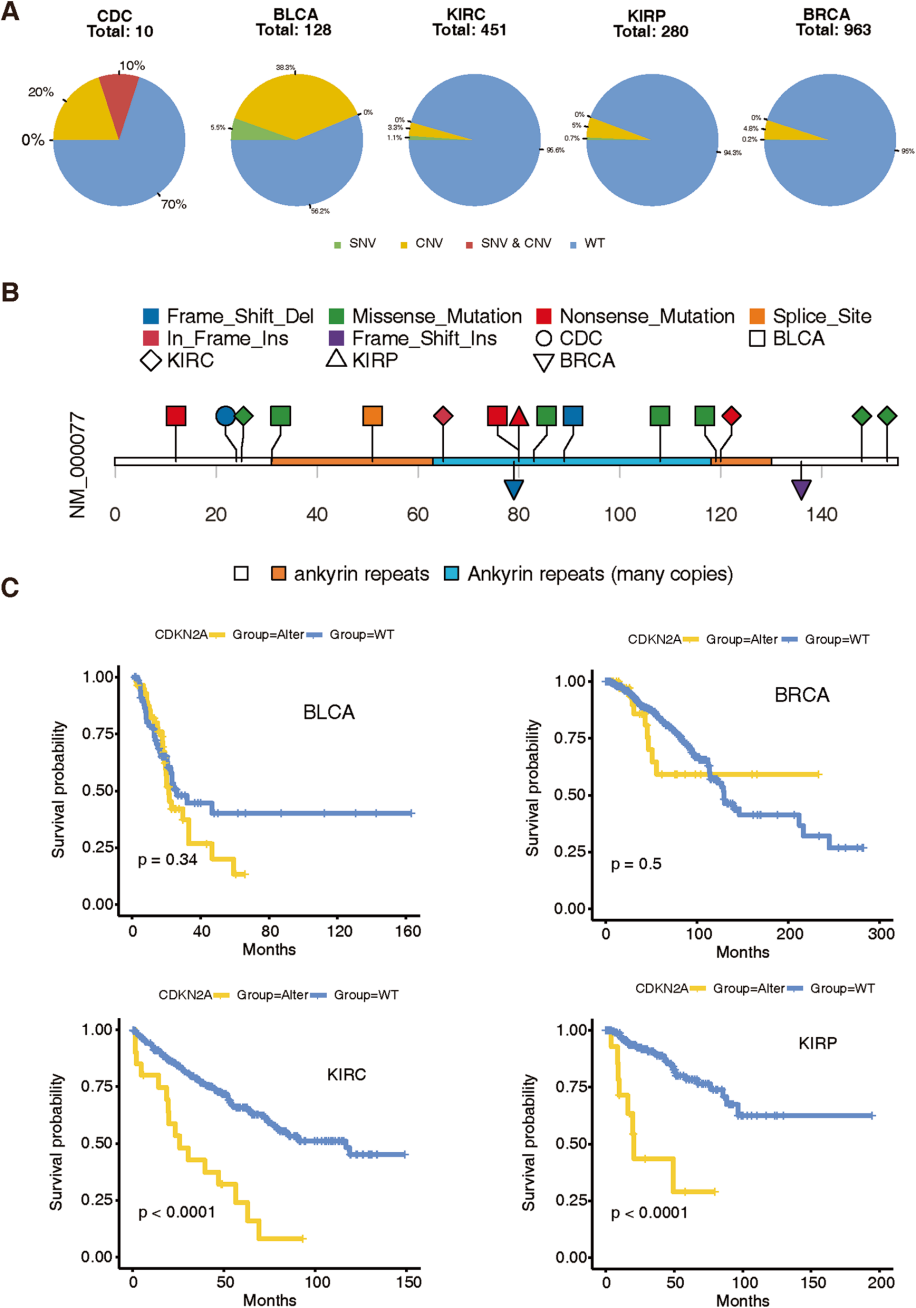
*CDKN2A* alteration is important in CDC and associated TCGA cohorts. **A** Components of single-nucleotide variants (SNV, green), copy number variants (CNV, orange), combinations of SNV and CNV (red), and wild-type (WT, blue) CDKN2A in different kinds of carcinomas (from left to right: CDC, BLCA, KIRC, KIRP and BRCA, respectively). **B** Lollipop plots of *CDKN2A* somatic variant mutation in different kinds of carcinomas. **C** Overall survival of patients with BLCA, BRCA, KIRC and KIRP between *CDKN2A*-altered and wild-type (WT) groups. KIRC, clear cell renal cell carcinoma; KIRP, papillary renal cell carcinoma; BLCA, bladder carcinoma; CDC, collecting duct carcinoma; BRCA, breast cancer

There was a higher percentage of CNV alterations than SNV alterations of *CDKN2A* in each cohort. The percentages of somatic mutated SNVs in CDC, BLCA, KIRC, KIRP and BRCA were 10%, 5.5%, 1.1%, 0.7% and 0.2%, respectively (Fig. 3A). Surprisingly, the *CDKN2A* mutation in our study, which encodes a protein change of p. R24Gfs*16 on exon 1, did not overlap with the previously reported *CDKN2A* mutation spectrum in urinary carcinomas and BRCA (Fig. 3B).

Moreover, patients with *CDKN2A* alteration displayed a significantly worse overall survival than patients with the wild-type gene in both the KIRC and KIRP cohorts. Despite no significant differences between *CDKN2A*-altered and wild-type cases in the BLCA and BRCA cohorts, these patients exhibited the same tendency in the early follow-up period (Fig. 3C). Overall, clinical data on *CDKN2A* in CDC are lacking due to its rarity.

### The *CDKN2A*-mediated p53/RB1 pathway is mostly altered in the CDC population

**Fig. 4:**
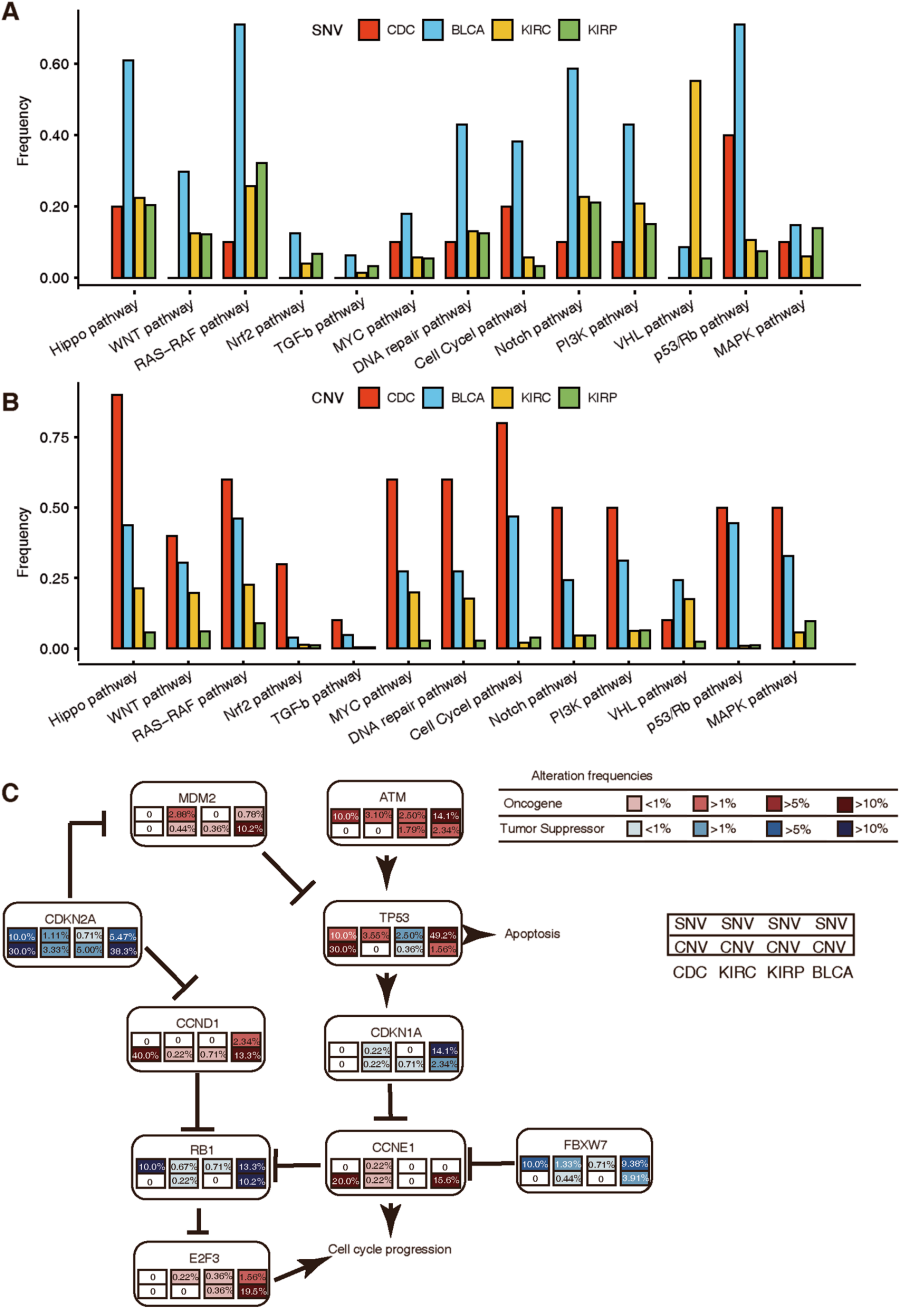
The *CDKN2A*-mediated p53/RB1 pathway is mostly altered in the CDC population. **A** Comparison of pathway-level alterations across the CDC, BLCA, KIRC and KIRP cohorts considering single-nucleotide variants (SNVs). **B** Comparison of pathway-level alterations across the CDC, BLCA, KIRC and KIRP cohorts considering copy number variants (CNVs). **C** SNVs and CNVs in components of the p53/RB1 pathway among the CDC, KIRC, KIRP and BLCA cohorts (from left to right in the box). Red, oncogene alterations; blue, tumour suppressor alterations. Percentages denote altered fractions. KIRC, clear cell renal cell carcinoma; KIRP, papillary renal cell carcinoma; BLCA, bladder carcinoma; CDC, collecting duct carcinoma

Genomic alterations are known to target common cancer pathways, even though not all component genes are altered at an equal frequency [27]. Next, we compared the mutational landscape among the CDC, KIRC, KIRP and BLCA cohorts in a pathway-centric manner. We focused on 10 important common pathways and three cancer-specific pathways based on both the SNV and CNV data, which suggested that the overall pathway-level mutation burden was different among the four cohorts (Fig. 4A, B; Additional file 10 and 11: Table S6 and S7).

Notably, the most frequently altered cancer-specific pathway in both the CDC and BLCA cohorts was the p53/RB1 pathway (Fig. 4C). However, individual altered genes varied significantly. In our CDC cohort, *CDKN2A* was most frequently altered, whereas *TP53* was the major mutated gene altered in the BLCA cohort. In addition, genes in the p53/RB1 pathway in the BLCA cohort were altered to different degrees. The KIRC cohort of TCGA typically showed specific VHL pathway alteration (Additional file 12: Fig. S5), which offers clinical drug targets, such as sunitinib and pazopanib. None of the key genes in the VHL pathway were altered in our CDC cohort, which might help to explain the limited efficacy of target therapies in CDC patients. In addition, *MET* was most frequently mutated in the specific MAPK pathway in the KIRP cohort but not in our CDC cohort (Additional file 13: Fig. S6).

Taken together, the *CDKN2A* alteration-mediated p53/RB1 pathway is most common in the CDC population, which might offer new insight into the clinical treatment of CDC patients.

## Discussion

In this study, we identified eight recurrently somatically mutated genes: *RBM14*, *MTUS1*, *GAK*, *DST*, *ASPM*, *CDC27*, *RNF213* and *XIRP2*. Except for *ASPM* and *CDC27,* six of these genes were validated by Sanger sequencing. In terms of the biological functions of these six genes [28–35], surprisingly, only *RBM14* and *MTUS1* have been reported to be associated with urothelial carcinomas. *RBM14* encodes a ribonucleoprotein that functions as a general nuclear coactivator and an RNA splicing modulator in a PARP-dependent DSB repair process [29]. *MTUS1*, a tumour-suppressor gene encoding angiotensin-II type 2 receptor-interacting proteins, is downregulated in clear cell renal cell carcinoma [31]. However, whether these mutations alter the clinical treatment of CDC patients remains unclear. Hence, these six genes might be new targets for CDC molecular therapy.

For the remaining mutated genes shown in Fig. 1, *TP53* is a known tumour-suppressor gene. *SETD2* was altered in both CDC and RCC subtypes but not in BLCA. Additionally, *CDKN2A* was a driver gene in both the CDC and BLCA but not in other RCC subtypes. In one patient in our study, arotinib target therapy achieved good efficacy after gemcitabine plus cisplatin chemotherapy. Taken together, these results suggest that CDC might present some shared therapeutic target molecules with other RCC subtypes and BLCA, which offers new insight into the systemic treatment of CDC patients.

Next, when we compared copy number variants in our CDC cohort with other subtypes of renal cell carcinomas and bladder urothelial carcinoma and found a unique mutation spectrum. In total, 29 amplified and 6 deleted recurrent focal CNVs were identified. In terms of driver genes in Cancer Gene Census, oncogenes *SKI*, *MUC4*, *TLX3*, *NPM1*, *KAT7*, *H3F3B*, *GNA11*, *MAP2K2*, *FSTL3*, *SH3GL1* and *U2AF1* and tumour-suppressor genes *RAD51B*, *DICER1*, *BCL11B*, *CCNB1IP1* and *BAZ1A* were detected. However, none of these genes are reported in association with renal cell carcinoma or urothelial carcinoma.

Notably, *CDKN2A,* a typical tumour-suppressor gene, was the shared gene in these four cohorts. Afterwards, we investigated the mutation spectrum of *CDKN2A*. *CDKN2A* encodes p16 and p14 to inactivate p53 [36]. Recently, a study reported 12.5% heterozygous losses and half homozygous losses in 16 patients with CDC [15]. Similar results were identified in 12% of 17 CDC patients, including one homozygous deletion and one truncation [13]. We also explored the role of *CDKN2A* in different subtypes of RCC, BLCA and BRCA. We detected a frameshift-deleted *CDKN2A* mutation in our CDC cohort, which suggested a novel mutated site different from any of the previously reported *CDKN2A* mutated sites in other urinary carcinomas and BRCA [37–40]. Furthermore, the most frequently cancer-specific altered pathway of this Chinese CDC cohort was the p53/RB1 pathway, which was consistent with our finding of *CDKN2A*. In addition, *CDKN2A*-altered patients displayed a significantly worse OS than patients without alterations in both the KIRC and KIRP cohorts, as validated by Girgis et al. [41], who found that KIRC with biallelic inactivation of *CDKN2A* indicates a poor prognosis. Importantly, CDKN2A upregulates expression of cyclin-dependent kinase 4 (*CDK4*)/*CDK6* [15], the corresponding selective inhibitors of which, such as palbociclib, ribociclib, and abemaciclib, interfere with cell cycle progression, induce cell senescence, and promote cancer cell disruption through a cytotoxic CD8+ T cell-mediated effect [22]. No *CDKN2A* alteration was found to correlate with clinical outcome in patients with platinum-refractory metastatic urothelial carcinoma, and palbociclib did not show meaningful clinical efficacy [42]. Moreover, *CDKN2A* loss is not associated with further benefit from palbociclib in combination with letrozole in the Palbociclib Ongoing Trials in the Management of Breast Cancer (PALOMA)-1 trial for patients with advanced *ER*+/*HER2*− breast cancer [43]. Nevertheless, a preclinical study of RCC cell lines reported that decreased *CDKN2A* is associated with sensitivity to *CDK4/6* inhibitors [44], with an effective response in KIRC with wild-type *VHL* and *CDKN2A* mutations due to palbociclib [45]. Pal et al. [46] also observed a meaningful response to palbociclib in a patient with metastatic CDC harbouring a *CDKN2A* homozygous deletion, which suggests a direction for further study of CDC patients. Taken together, our findings indicate that this *CDKN2A* alteration-mediated pathway represents a rational and novel therapeutic strategy target for CDC, even for all urothelial carcinomas, which needs further validation in patients.

There are some limitations to our study. First, this was a single-centre, retrospective study with a small sample size due to the rare incidence of the cancer. Second, it was a study of single omics, which might also lack sufficient validation information. Third, there was a lack of collecting duct carcinoma cell lines and animal models to complete validations in vivo and in vitro. Hence, in the future, we will conduct a prospective, randomised and multicentre clinical trial to obtain multi-omics data to validate our findings.

## Conclusion

In conclusion, our study offers a genomic spectrum of a Chinese population with CDC. CDC is not only characterised as a unique type of solid tumour, but it shares some specific molecules with other RCC subtypes and urinary tract carcinoma. The *CDKN2A* alteration-mediated p53/RB1 pathway might provide new insight into potential prognostic and therapeutic targets for CDC patients.

## Supplementary Information

**Additional file 1: Table S1.** The summary of the clinicopathological features of the patients included.

**Additional file 2: Table S2.** The summary of the whole exosome sequencing data in patients with CDC.

**Additional file 3: Table S3.** The summary of the filtered somatic short variant discovery.

**Additional file 4: Figure S1.** The Sanger validations of *RBM14*, *GAK*, *DST* and *XIRP2*.

**Additional file 5: Figure S2.** The Sanger validations of *SETD2*, *MTUS1*, *RNF213*, *ASPM* and *CDC27*.

**Additional file 6: Figure S3.** The mutation signature of collecting duct carcinoma patients in our study.

**Additional file 7: Table S4.** GISTIC2.0 results of deleted genes in CDC, BLCA, KIRC and KIRP cohorts.

**Additional file 8: Figure S4.** Somatic copy number variants (CNV) detection.

**Additional file 9: Table S5.** GISTIC2.0 results of amplified genes in CDC, BLCA, KIRC and KIRP cohorts.

**Additional file 10: Table S6.** Details of pathway-level alterations across the CDC, BLCA, KIRC and KIRP cohorts considering the single nucleotide variants (SNV).

**Additional file 11: Table S7.** Details of pathway-level alterations across CDC, BLCA, KIRC and KIRP cohorts considering the copy number variants (CNV).

**Additional file 12: Figure S5.** The mutation landscape on the KIRC specific pathway.

**Additional file 13: Figure S6.** The mutation landscape on the KIRP specific pathway.

## Data Availability

The raw sequence data reported in this paper have been deposited in the Genome Sequence Archive in National Genomics Data Center, China National Center for Bioinformation/Beijing Institute of Genomics, Chinese Academy of Sciences, under accession number HRA001344 that are publicly accessible at https://ngdc.cncb.ac.cn/gsa-human (https://ngdc.cncb.ac.cn/gsa-human/browse/HRA001344).

## Abbreviations

BLCA: Bladder urothelial carcinoma
BRCA: Breast cancer
CDC: Collecting duct carcinoma
CGC: Cancer gene census
CNV: Copy number variant
COSMIC: Catalogue of somatic mutations in cancer
EAS: East Asian
ESP: Exome sequencing project
FFPE: Formaldehyde-fixed paraffin-embedded
GETUG: Groupe d'Etudes des Tumeurs Uro-Génitales
IGV: Integrative genomics viewer
NCCN: National comprehensive cancer network
ORR: Objective response rate
OS: Overall survival
RCC: Renal cell carcinoma
SNV: Single-nucleotide variant
TCGA: The cancer genome atlas
UTUC: Upper tract urothelial carcinoma
WES: Whole-exome sequencing
WT: Wild-type
