# Supplementary figures and images for: A genomic mutation spectrum of collecting duct carcinoma in the Chinese population
Source: BMC Med Genomics. 2022 Jan 3;15:1. doi: 10.1186/s12920-021-01143-2 (PMC8722201; doi:10.1186/s12920-021-01143-2)

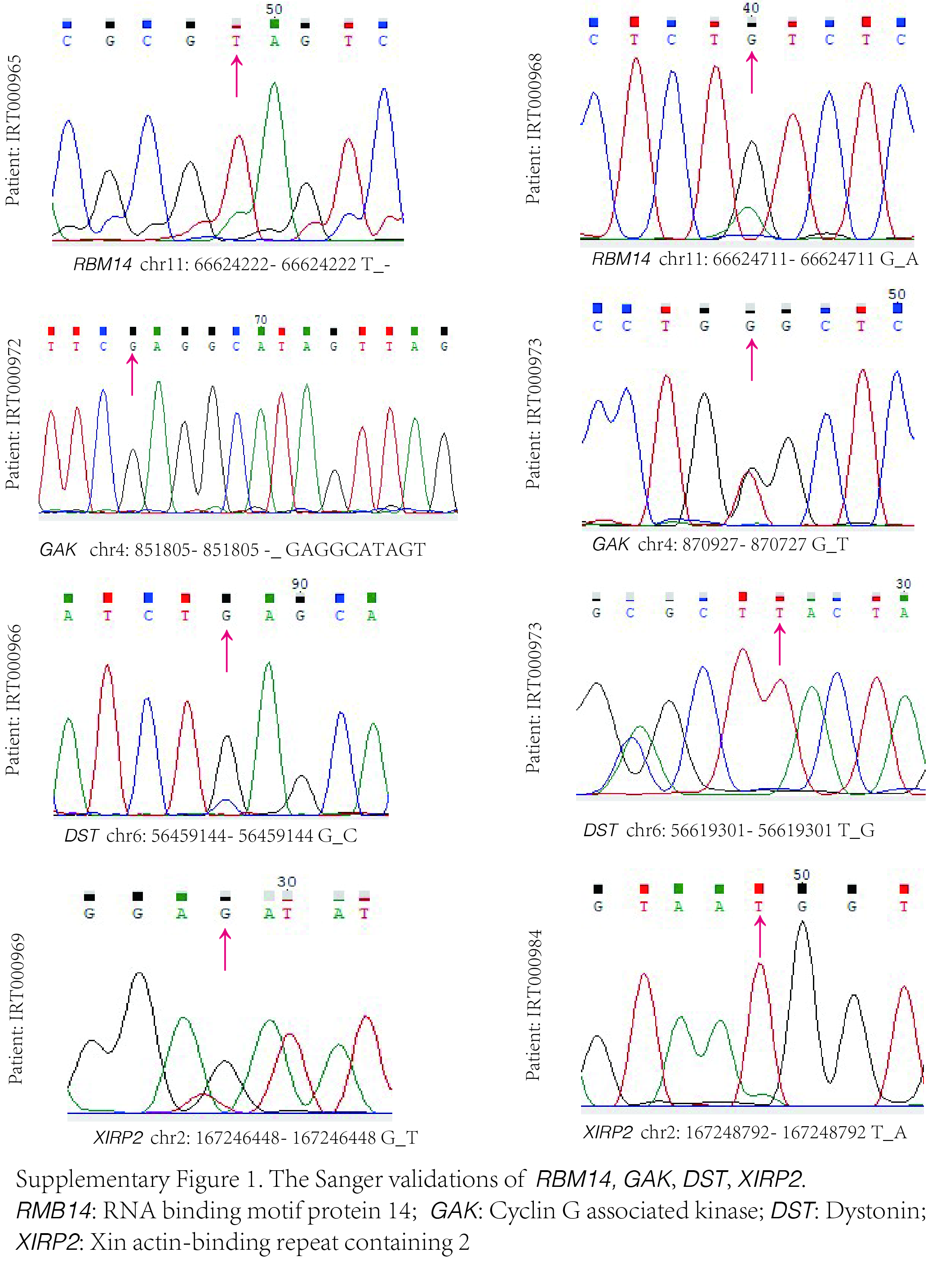

Supplement: Supplementary file 4 — Additional file 4: Figure S1. The Sanger validations of RBM14, GAK, DST and XIRP2. [file 12920_2021_1143_MOESM4_ESM.tif]

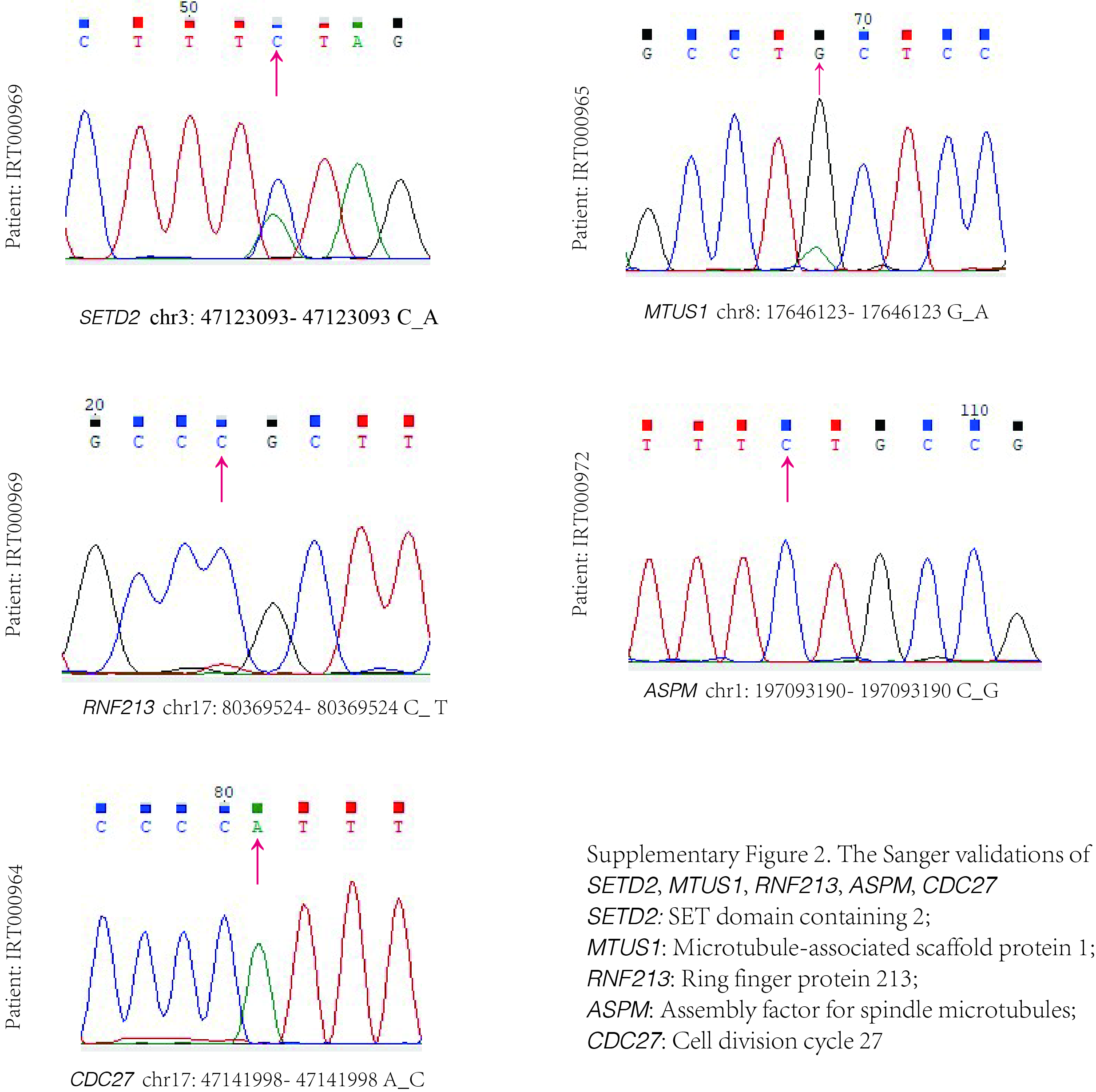

Supplement: Supplementary file 5 — Additional file 5: Figure S2. The Sanger validations of SETD2, MTUS1, RNF213, ASPM and CDC27. [file 12920_2021_1143_MOESM5_ESM.tif]

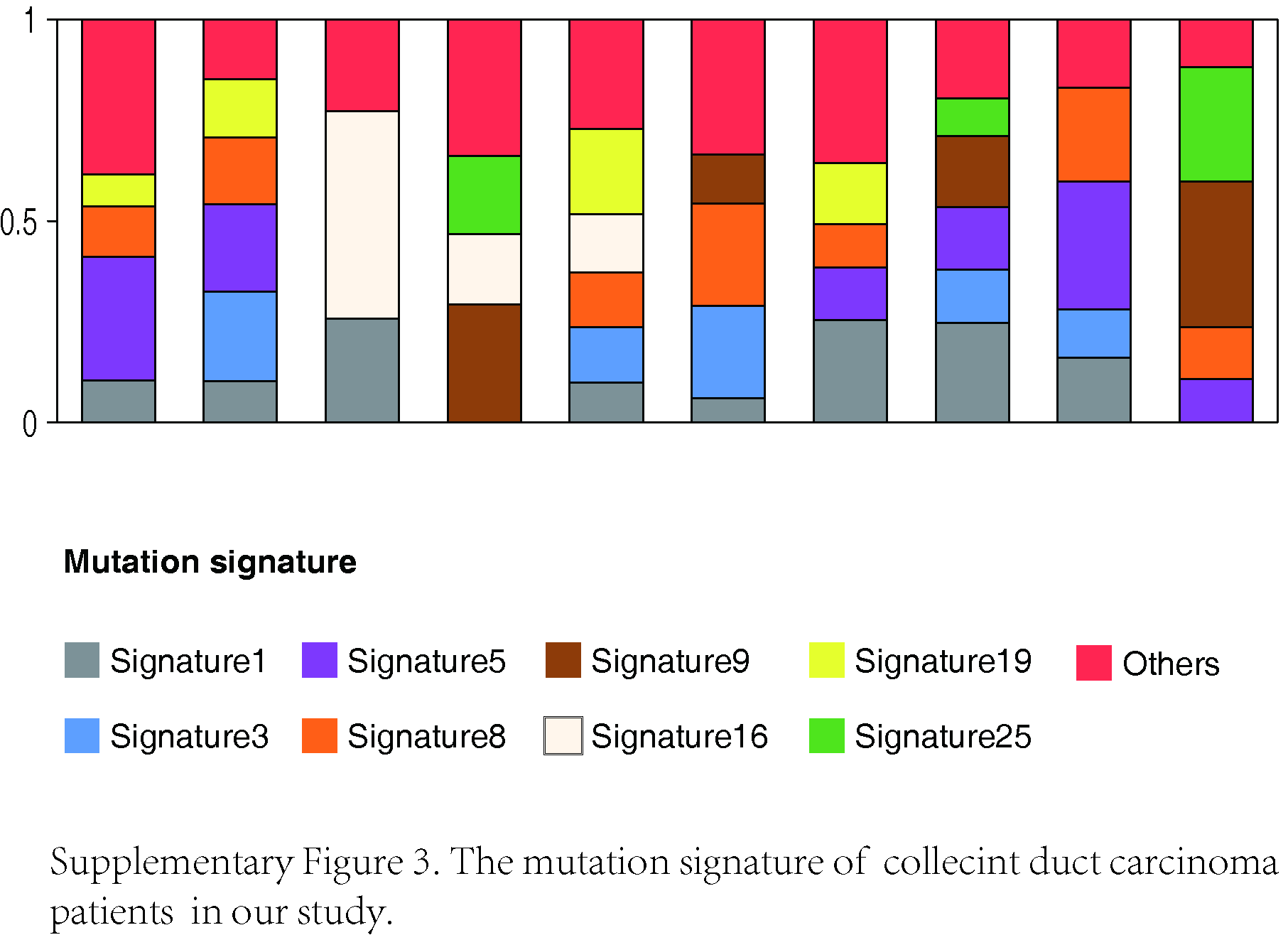

Supplement: Supplementary file 6 — Additional file 6: Figure S3. The mutation signature of collecting duct carcinoma patients in our study. [file 12920_2021_1143_MOESM6_ESM.tif]

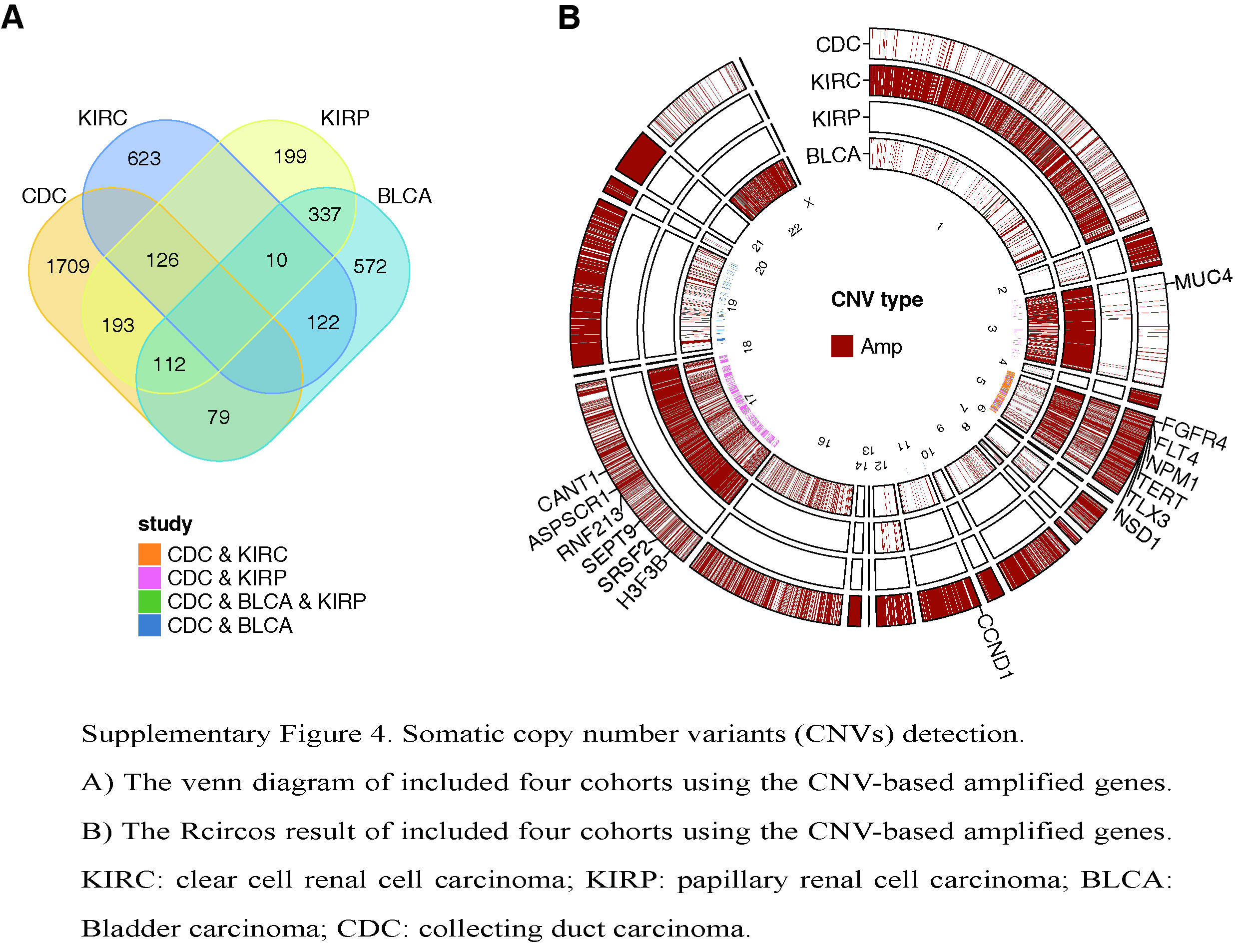

Supplement: Supplementary file 8 — Additional file 8: Figure S4. Somatic copy number variants (CNV) detection. [file 12920_2021_1143_MOESM8_ESM.tif]

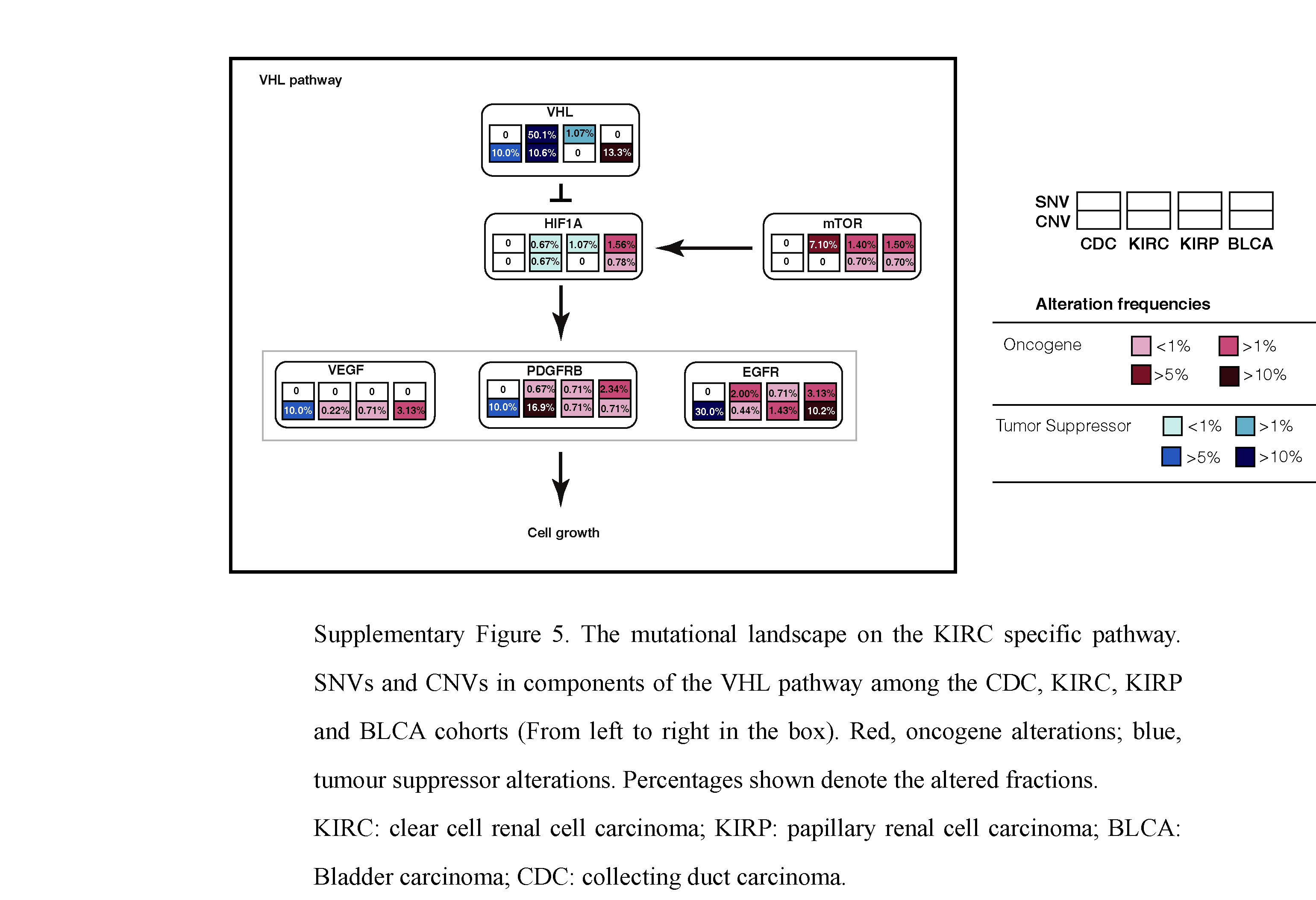

Supplement: Supplementary file 12 — Additional file 12: Figure S5. The mutation landscape on the KIRC specific pathway. [file 12920_2021_1143_MOESM12_ESM.tif]

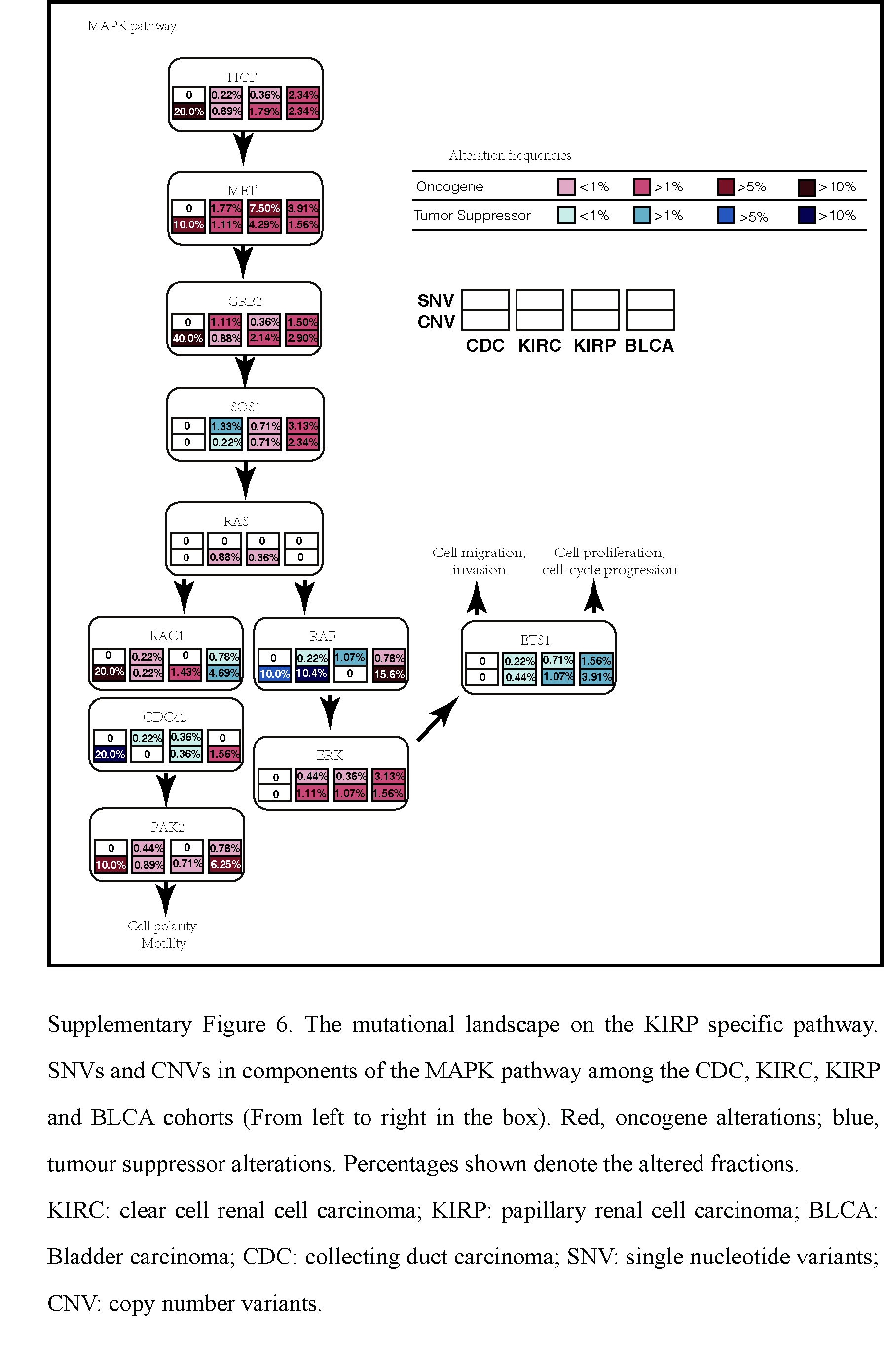

Supplement: Supplementary file 13 — Additional file 13: Figure S6. The mutation landscape on the KIRP specific pathway. [file 12920_2021_1143_MOESM13_ESM.tif]
